# Relationship between Female Sexual Dysfunction and Trunk Stability Post-Stroke: A Cross-Sectional Study

**DOI:** 10.3390/medicina60020324

**Published:** 2024-02-14

**Authors:** Irene del Mar Robledo-Resina, Carlos Romero-Morales, Patricia Martín-Casas, Jorge Hugo Villafañe, Vanesa Abuín-Porras

**Affiliations:** 1 Department of Physiotherapy, Faculty of Sport Sciences, Universidad Europea de Madrid, 28670 Villaviciosa de Odón, Spain; irene_resina@hotmail.com (I.d.M.R.-R.); carlos.romero@universidadeuropea.es (C.R.-M.);; 2 Department of Radiology, Rehabilitation and Physiotherapy, Faculty of Nursing, Physiotherapy and Podiatry, Complutense University of Madrid, 28040 Madrid, Spain; pmcasas@ucm.es; 3 Musculoskeletal Pain and Motor Control Research Group, Faculty of Sport Sciences, Universidad Europea de Madrid, 28670 Villaviciosa de Odón, Spain

**Keywords:** stroke, sexual dysfunction, trunk stability

## Abstract

*Background and Objectives*: Stroke can lead to a variety of consequences, the severity and nature of which are contingent upon the affected brain region or lesion type. These consequences manifest with distinct clinical presentations and recovery trajectories. This study aims to investigate the potential correlation between feminine sexual dysfunction and trunk stability among stroke survivors. *Materials and Methods*: Thirty-eight women (stroke group *n* = 19 and control group *n* = 19) were recruited. A cross-sectional observational study was designed. Outcome measures were recorded using the Feminine Sexual Function Index, the National Institute of Health Stroke Score, the Newcastle Stroke-specific Quality of Life Measure Beck Depression Index, the Barthel Index, the Urge-urinary Distress Inventory, and the Trunk Impairment Scale. Spearman’s correlation was tested between different factors influencing feminine sexual dysfunction and trunk stability. *Results*: Statistically significant differences were found in sexual function between the stroke group versus the control group (Z = 88; *p* = 0.007; rb = 0.51). The correlation showed a relationship between feminine sexual dysfunction and trunk stability (*p* < 0.05). A relationship between quality of life and sexual dysfunction was also found (*p* < 0.05). There were no statistically significant results for the association between dependency, severity of stroke, time after stroke type of stroke, and sexual dysfunction (*p* = 0.378). *Conclusions*: The results of this study support the existence of a correlation between feminine sexual dysfunction and trunk stability, probably due to trunk and pelvic floor muscle synergy. Multidisciplinary teams assessing sexual dysfunction after stroke should include a physical therapist to assess the physical components that may interfere with feminine sexual health post-stroke.

## 1. Introduction

Stroke is one of the leading causes of disability and the second primary cause of mortality in Western Countries, with an increasing prevalence in young people and women [1]. Consequences after suffering a stroke can differ, depending on the injured area or the lesion type, with different clinical expressions and recovery profiles [2].

The consequences of stroke extend beyond physical impairment, impacting various interconnected aspects of life including social, emotional, and sexual well-being [3,4]. Amongst these sequels, pelvic floor alterations are frequently reported, leading to sexual dysfunction (SD). Regrettably, conventional stroke rehabilitation programs typically prioritize functional aspects of daily living, neglecting the crucial dimension of sexuality as a determinant of overall quality of life [5]. However, individuals with central nervous system conditions, including stroke survivors, disproportionately experience SD compared to those with other chronic ailments, with reports indicating that 20 to 75% of stroke patients encounter changes in sexual function post-stroke [6]. Furthermore, there exists a paucity of research specifically addressing female sexuality in the context of stroke recovery, with the majority of studies predominantly focused on male experiences [4,5,7].

Female sexual health is often neglected, especially in patriarchal societies, despite its significant impact on women’s overall well-being. In addition to physiological factors, emotional, physical, and social aspects contribute to maintaining sexual health. Despite being underreported, female sexual dysfunction is prevalent and involves a complex interplay of psychosocial and physiological factors [8]. Female sexual dysfunction encompasses a spectrum of issues spanning arousal, sexual desire, orgasm, and post-coital pain [9,10,11]. The literature about stroke patients indicates a decrease in the sexuality-related life quality in this population, reporting libido decrease, arousal problems, orgasmic dysfunction, lack of vaginal lubrication, and pain during sexual activity. Patients usually report structural barriers, such as pain, limited mobility, altered muscular tone (i.e., spasticity), sensory alterations, fatigue, and urinary or fecal incontinence, that interfere with sexual activity. In addition, the influence of psycho-emotional and social aspects—depression, anxiety, fear, changes in self-perception, and changes in the couple’s role—has a direct impact on sexual quality in women post-stroke. Post-stroke rehabilitation focused on SD traditionally targets psychological characteristics, disregarding anatomical and physical aspects that can interfere with sexual function [12,13,14,15].

Urinary incontinence (UI) poses significant challenges to women’s quality of life and health, leading to mental–social disorders such as depression, reduced self-confidence, and limitations in daily activities [16]. The intricate interplay between pelvic floor muscles and trunk stability is increasingly recognized in the realm of rehabilitation. Pelvic floor muscles function in tandem with the abdominal wall, diaphragm, deep trunk muscles, and lumbar musculature to facilitate visceral support, urinary and fecal continence, and maintain postural stability against gravitational forces [17,18,19]. In addition, pelvic floor muscles seem to be closely related to abdominal wall muscles, such as the diaphragm, deep muscles of the trunk, and lumbar area, acting as a synergic unit for sensory–motor function and stability [16,20]. Consequently, any dysfunction within the pelvic floor musculature has the potential to disrupt the integrity of the abdominal–pelvic–lumbar muscular complex, thereby compromising trunk stability, and vice versa. Emerging evidence underscores the symbiotic relationship between pelvic floor and trunk muscle activation, emphasizing the therapeutic benefits of concurrent training of these muscle groups in optimizing functional outcomes [21].

This study aims to elucidate the correlation between female sexual dysfunction following stroke and trunk stability. We postulate that a significant association exists between these variables, given the synergistic role of the pelvic floor and trunk muscles in maintaining trunk stability. Furthermore, we explore the influence of various known determinants—both psychological and physical—on post-stroke SD, including perceived quality of life, depressive symptoms, stroke severity, and urinary incontinence, with the aim of comprehensively understanding the multifactorial nature of sexual dysfunction in stroke survivors.

## 2. Materials and Methods

### 2.1. Study Design

A case–control, observational, cross-sectional study was designed following the STROBE (Strengthening the Reporting of Observational Studies in Epidemiology) guidelines [22].

### 2.2. Ethical Considerations

This study followed the ethical standards of the Declaration of Helsinki and was revised and approved by the ethics committee. Each participant signed the informed consent form before entering the study. This study received no funding.

### 2.3. Participants

Subjects from the case group were recruited in a Stroke and Brain Injury specialized Spanish Hospital, from January to March 2022. The control group (CG) was recruited in matching age ranges with the stroke group (StrG) (StrG mean 50 ± 10.5 years old/CG mean 44.8 ± 12.8 years old). Inclusion criteria for the StrG were stroke diagnosis in the past 12 months and aged 20 to 65 years old. For the CG the criteria were the absence of central nervous system pathology and aged 20 to 65 years old. Exclusion criteria for the StrG were cognitive impairment that interfered with the understanding or performing of the study proceedings and aphasia.

### 2.4. Outcome Measures

Clinical data regarding SD were collected through the Feminine Sexual Function Index (FSFI), a questionnaire with 19 items analyzing 7 areas, desire, arousal, libido, lubrication, orgasm, satisfaction, and pain, with a maximum value of 36, indicating absence of SD, and a minimum value of 2 [23]. The questionnaire can be used to assess changes in quality of life with regard to sexuality.

Trunk stability (TS) was measured through the Trunk Impairment Scale (TIS 2.0). TIS evaluates 14 items divided into two categories, balance and trunk coordination in a sitting position, with final calculated values rating from 0 to 1, with 1 considered as total trunk stability [24].

Stroke severity was recorded by the National Institute of Health Stroke Score (NIHSS), which classifies severity into no stroke symptoms (0), minor (1–4), moderate (5–15), moderate to severe (16–20), and severe (21–42). The dependency level was assessed with the Barthel Index (BI). Guidelines for interpretation state that results of 0–20 indicate total dependency, 21–60 severe, 61–90 moderate, and 91–99 indicate “slight” dependency. Newcastle Stroke-specific Quality of Life Measure (NEWSQOL) was used to analyze the quality of life, ranging from 0 to 100. A maximum score indicates high life quality. The Beck Depression Index (BDI-II) was selected to evaluate depressive symptoms. Values range from 0 to 84, divided into 21 Items. High values are considered an indication of depressive symptomatology. Finally, urinary incontinence was assessed by the Urge-urinary Distress Inventory (UDI-6), a descriptive questionnaire that considers all types of urinary incontinence, ranging from 0 (no incontinence) to 18 (incontinence).

Other variables, including stroke type, time since stroke, age, height and weight, were recorded in an initial interview.

Subjects were asked to answer the questionnaires by themselves, in a closed room, with a minimum time of 30 and a maximum of 40 min to complete the tests. In addition, a physical therapist was in charge of the recording of the demographic data and the evaluation of the TS by the TIS 2.0. Each subject was registered with a randomly generated number.

### 2.5. Sample Size

Sample size calculation was carried out through a pilot study with 10 subjects per group, using G*Power 3.1.9.2 software, applying the main outcome, sexual dysfunction (mean ± Standard Deviation): 10 StrG (16.44 ± 8.29) and 10 CG (25.03 ± 4.32). For the sample size calculation, a 1-tailed hypothesis, an α error probability of 0.05, a power (1-β error probability) of 0.95, and an allocation ratio (N2/N1) of 1 were utilized. Thus, a total sample size of 28 subjects, 14 for each group, was calculated. Considering 15% of possible participants lost to follow-up, a total sample of 38 subjects, 19 in each group, was considered. A post hoc analysis to calculate achieved power was performed for the SD variable, obtaining a 0.95 value.

### 2.6. Statistical Analysis

All statistical analyses were performed with R-based software jamovi V.2.0, Sydney, Australia (www.jamovi.org, accessed on 14 October 2023), with an α error of 0.05 (95% confidence interval), a desired power or 1−β = 0.95. According to Shapiro–Wilk test results (*p* < 0.05) variables SD, TS, IU, QL, and DS were analyzed through non-parametric tests (Mann–Whitney *U* and Spearman correlation). The effect size for these variables was calculated with Rank Biserial Correlation, with values of r_b_ = 0.10, 0.30 and 0.50 for low, medium, and high, respectively. For the rest of the variables, parametric tests were used (Pearson). To quantify which variables could better explain the presence of SD, a linear regression analysis was performed, taking into account explained variation (R^2^), collinearity, and autocorrelation (Durbin–Watson), with an α value of 0.05. An incidence study was performed for variables ACV type and time from ACV using frequency analysis (χ^2^ test).

## 3. Results

Regarding age, the StrG displayed a mean age of 50.0 years, with a median of 52 years, while the CG showed a mean age of 44.8 years, with a median of 50 years. In terms of weight, the StrG had an average of 66.0 kg, whereas the CG exhibited an average of 66.5 kg. Average height was similar between groups, with 1.7 m for StrG and 1.6 m for CG. Regarding BMI, the StrG had an average of 24.0, whereas the CG showed an average of 25.0. Concerning QL, the StrG presented an average score of 62.8, whereas the CG displayed a significantly lower average score of 20.1. As for depressive symptoms (DS), the StrG showed an average of 16.1, while the CG exhibited an average of 12.1. Regarding UI, both groups had similar scores, with an average of 19.7 for StrG and 19.7 for CG.

Results from descriptive analysis of both groups are shown in Table 1. All the variables analyzed showed statistically significant differences in intergroup comparison except for depressive symptoms and urinary incontinence, (Z-value = 128.50; *p* = 0.132; r_b_ = 0.29) and (Z-value = 175.50; *p* = 0.895; r_b_ = 0.03), respectively.

Regarding SD modalities including desire, arousal, libido, lubrication, orgasm, satisfaction, and pain, there were statistically significant differences between groups, except for the pain modality (Table 2). A Mann–Whitney U test revealed significant differences between the SG and the CG across all domains except for pain (*p* = 0.150). Effect sizes, as indicated by rank biserial correlation coefficients, ranged from moderate to large, with values of 0.27 to 0.64, suggesting substantial practical significance.

Furthermore, a significant negative correlation was observed between SD and quality of life (*p* = 0.006, rho = −0.44). However, the predictive power of quality of life for SD depending on the group (SG or CG) was not significant according to the adjusted linear graphic.

Spearman correlation analysis between sexual dysfunction and trunk stability revealed statistically significant (*p* < 0.05) and positive (rho = +0.81) results, indicating a strong correlation. This implies that lower values in the Feminine Sexual Function Index, indicative of SD, are associated with lower values in the Trunk Impairment Scale, reflecting poor TS. This correlation was consistently observed across all SD modalities, including desire, arousal, libido, lubrication, orgasm, satisfaction, and pain (Table 3).

This strong correlation underscores the intricate relationship between sexual function and trunk stability in stroke patients. It suggests that individuals with diminished trunk stability may experience greater sexual dysfunction, likely due to physical limitations that affect their ability to engage in sexual activities comfortably.

Furthermore, a linear regression model was employed to explain SD through TS, revealing that the relationship is group dependent (Table 4). The intersection point, sexual dysfunction estimators, and *p*-values indicate that trunk stability significantly influences sexual dysfunction, and this influence varies between the StrG and the CG. This finding highlights the importance of considering trunk stability when assessing and addressing sexual function in stroke patients, as it may serve as a key factor in understanding and managing sexual dysfunction in this population.

Moreover, the severity of stroke and dependency, as well as the type of stroke and time since stroke, did not yield statistically significant results in their correlation with SD (*p* = 0.286; r = −0.26 and *p* = 0.137; r = 0.35, respectively). Similarly, there were no statistically significant differences in the frequency of SD related to the type of stroke or time since stroke, according to the χ^2^ test (*p* = 0.378 and *p* = 0.328, respectively). These results suggest that factors other than stroke severity, dependency, type, or time since stroke may play a more prominent role in influencing sexual dysfunction in stroke patients. Further research is warranted to explore these factors comprehensively.

## 4. Discussion

The results of our study offer significant insights into the complex relationship between post-stroke SD and TS, shedding light on various facets of both conditions and their intricate interplay. Our investigation aimed to explore this relationship, hypothesizing that the coordination between PF and trunk muscles plays a pivotal role in linking these two phenomena. The findings from our study indeed support this hypothesis, revealing a consistent association between SD and TS, which aligns with previous research investigating the interaction between effective activation of the abdomino–lumbar–pelvic complex and its impact on PF function. Our descriptive analysis of the study groups provided a comprehensive overview, highlighting significant differences between the StrG and the CG across various variables. While variables such as depressive symptoms and urinary incontinence did not exhibit significant differences between groups, others such as SD, QL, and TS showed notable distinctions. This underscores the multifactorial nature of post-stroke sexual function, emphasizing the importance of considering various factors in its assessment and management [25,26]. Sapsford et al. (2001, *n* = 7 subjects) [27] reported in their study the existence of a coactivation pattern, assessed in supine between PF muscles and abdominal muscles. In their studies, Capson et al. (2011, *n* = 16 subjects) [21] and Neumann et al. (2002, *n* = 4 subjects) [28] also pointed out the existent synergy between PF and trunk muscles in standing positions, emphasizing the influence of posture in the activation pattern. Nevertheless, a recent study by Ko M et al. (2022, *n* = 20 subjects) [16] reported that the activation of PF muscles in a sitting position creates an inconsistent pattern of activation response in trunk muscles, concluding that they may be more involved in maintaining a position against gravity than in acting as coactivators of PF muscles [29]. A possible reason for the discrepancy in this last study’s results could be the difference in the assessment position, as trunk and PF muscles can plausibly change their coactivation pattern depending on postural demands [30]. Thus, more studies are needed, both in healthy subjects and in populations with impaired postural control, to determine the exact relation between those muscular groups in order to guide interventions [31].

Moreover, in this study, severity and dependence post-stroke surprisingly did not have a strong influence on SD. These results show the multifactorial nature of the feminine sexual function and the necessity of a multidisciplinary intervention to assess this item as a part of the quality-of-life concept. Sexual function in the post-stroke group showed lower total values compared to healthy individuals. The most affected areas in StrG compared to CG were desire, arousal, lubrication, orgasm, and sexual satisfaction. These findings corroborate previous research indicating the impact of stroke-related sensory–motor alterations on sexual function [32]. Interestingly, while significant correlations were observed between SD and QL, the predictive power of QL for SD was not significant when considering the group factor, suggesting potential differences in the determinants of SD between stroke and control groups. In addition, StrG and CG showed similar, moderate incidences of depressive symptoms and urinary incontinence. It has to be pointed out that the mean age of StrG (50) and CG (44) is considered young, according to the scientific literature involving post-stroke SD and feminine SD in general [33,34]. These findings about the presence of depressive symptoms and urinary incontinence in the general population are not new in the scientific literature, revealing some challenges affecting sexual function that could be present in society, without any existence of stroke sequelae [35,36].

Additionally, our study shed light on the multifaceted nature of feminine sexual function post-stroke, with lower total values observed in desire, arousal, lubrication, orgasm, and sexual satisfaction domains compared to healthy individuals. These observations resonate with prior research attributing these differences to post-stroke sensory–motor alterations and specific factors related to arousal, orgasm, and pain [37].

Despite the advancements in addressing post-stroke sexual dysfunction, our findings underscore the challenges in addressing sexuality, especially in a disability context [38]. While sexual education remains a primary therapeutic avenue for post-stroke individuals, our study underscores the importance of considering physical factors such as trunk stability in these evaluations. Further research is warranted to elucidate underlying mechanisms and develop targeted therapeutic strategies to enhance sexual function in stroke survivors, ultimately improving their overall quality of life [39].

### 4.1. Clinical Implications

Our study revealed significant differences between stroke and control groups in SD, quality of life, and trunk stability. Importantly, we identified a strong positive correlation between SD and trunk stability, underscoring the need to address sexual dysfunction alongside physical recovery in rehabilitation programs. Multidisciplinary collaboration is essential in this regard, with tailored education interventions and comprehensive physical assessments to address both physical and psychosocial barriers to sexual function.

### 4.2. Limitations

Despite the insights gained from this study, several limitations need acknowledgment. Firstly, the challenge of accessing the specific target population of young women who have experienced stroke events posed significant difficulties. Additionally, there was a notable reticence among participants to openly discuss their sexuality, reflecting the sensitive nature of the topic and potential cultural taboos. For many participants, this study marked the first instance where healthcare professionals broached the subject of stroke sequelae’s impact on sexual function and its broader repercussions on personal life. This hesitancy to engage in discussions about sexuality may have influenced the depth and accuracy of the data collected, potentially leading to underreporting or misrepresentation of experiences.

Moreover, this study’s cross-sectional design limits the ability to establish causal relationships between variables. While correlations between sexual dysfunction and trunk stability were observed, causality cannot be inferred, and longitudinal studies are warranted to elucidate the temporal dynamics of these relationships over time. Additionally, the sample size, though sufficient for the statistical analyses conducted, may not fully capture the heterogeneity and complexity of post-stroke sexual dysfunction experiences among women. Larger sample sizes would enhance the generalizability and robustness of the findings, allowing for more nuanced subgroup analyses based on factors such as stroke severity, type, and time since onset.

Furthermore, the reliance on self-reported measures introduces the potential for response bias and social desirability effects, particularly concerning sensitive topics such as sexual function and mental health. Participants may underreport or overreport symptoms due to perceived stigma or societal expectations, leading to measurement inaccuracies. Future studies could benefit from incorporating objective measures, such as physiological assessments of pelvic floor function and trunk stability, to complement self-reported data and enhance the validity of findings.

Finally, the study’s recruitment from a single specialized hospital may limit the generalizability of results to broader populations of stroke survivors. Variations in healthcare access, cultural factors, and stroke management practices across different settings could influence the prevalence and presentation of sexual dysfunction post-stroke. Multi-center studies involving diverse populations are needed to validate and extend the findings of this study, providing a more comprehensive understanding of the factors contributing to post-stroke sexual dysfunction in women.

## 5. Conclusions

Our study highlights a significant correlation between female sexual dysfunction and trunk stability among patients recovering from stroke, emphasizing the need to address sexual dysfunction alongside physical rehabilitation efforts. The robust association observed suggests that individuals with compromised trunk stability may experience heightened sexual dysfunction, likely due to physical limitations hindering comfortable engagement in sexual activities. This underscores the importance of considering trunk stability as a pivotal factor when evaluating and managing sexual function in stroke patients.

Moreover, our research underscores significant disparities in SD, quality of life, and trunk stability between stroke and control groups, emphasizing the imperative of integrating sexual dysfunction management within stroke rehabilitation programs. This necessitates multidisciplinary collaboration, incorporating tailored educational interventions and comprehensive physical assessments to address both physical and psychosocial barriers to sexual function.

Despite the valuable insights garnered, we acknowledge several limitations. Accessing the specific demographic of young women who have experienced stroke events presented significant challenges. Additionally, participants’ reluctance to openly discuss their sexuality, possibly influenced by the sensitive nature of the topic and cultural taboos, may have impacted data depth and accuracy, potentially leading to underreporting or misrepresentation of experiences.

In summary, post-stroke rehabilitation programs should adopt holistic approaches to restore individuals physically, psychologically, and socially. Given the considerable negative impact of SD on quality of life, its inclusion in rehabilitation programs is crucial. These programs should adapt to the altered sexual environment and provide education interventions tailored to patients and their partners. Furthermore, multidisciplinary collaboration involving psychologists, occupational therapists, and physical therapists is essential. Particularly, physical therapy should encompass comprehensive assessments of physical barriers impeding sexual function, including TS and pelvic floor muscle function, while addressing sensory–motor impairments, spasticity, and mobility. Future research with larger sample sizes is imperative to unravel the intricate complexities of SD in women after stroke.

## Figures and Tables

**Table 1:** Descriptive Statistics.

	Group	Age	Weight	Height	BMI	SD	QL	DS	UI	TS
*n*	StrG	19	19	19	19	19	19	19	19	19
	CG	19	19	19	19	19	19	19	19	19
x̄	StrG	50.0	66.0	1.7	24.0	16.8	62.8	16.1	19.7	0.49
	CG	44.8	66.5	1.6	25.0	26.1	20.1	12.1	19.7	0.86
Me	StrG	52	69	1.7	22.0	17.1	62	15	16.7	0.50
	CG	50	65	1.7	25.0	27.2	18	8	20.8	0.88
DS	StrG	10.5	12.3	0.10	4.3	10.7	28.3	10.0	17.2	0.24
	CG	12.8	10.0	0.08	4.2	6.3	15.8	10.0	14.6	0.13
Min	StrG	30	49	1.51	18.4	2.6	17	3	0.00	0.13
	CG	24	52	1.50	18.7	6.8	0	0	0.00	0.56
Max	StrG	65	85	1.89	31.6	33.3	112	38	54.2	0.88
	CG	65	84	1.74	32.0	33.0	53	39	45.8	1.0
SW (w)	StrG	0.96	0.91	0.93	0.92	0.92	0.95	0.91	0.91	0.94
	CG	0.91	0.93	0.93	0.95	0.84	0.88	0.86	0.94	0.87
SW (*p*)	StrG	0.52	0.07	0.16	0.10	0.11	0.46	0.08	0.07	0.27
	CG	0.08	0.20	0.17	0.42	0.01	0.02	0.01	0.28	0.01

x̄= mean; Me = median; SD = standard deviations; Min = minimum values; Max = maximum values; SW = Shapiro–Wilk; StrG = stroke group; CG = control group. BMI = Body Mass Index; SD = Sexual Dysfunction; QL = Quality of Life; DS = Depressive Symptoms; UI = Urinary Incontinence and TS = Trunk Stability.

**Table 2:** Sexual dysfunction questionnaire domains. Intergroup comparison.

Question	Test	Statistic	*p*	Correlation Type	Effect Size
Desire	Mann–Whitney U	103.0	0.028	Rank biserial correlation	0.41
Arousal	Mann–Whitney U	100.5	0.020	Rank biserial correlation	0.44
Lubrication	Mann–Whitney U	108.0	0.035	Rank biserial correlation	0.40
Orgasm	Mann–Whitney U	65.0	<0.001	Rank biserial correlation	0.64
Satisfaction	Mann–Whitney U	88.5	0.007	Rank biserial correlation	0.51
Pain	Mann–Whitney U	131.5	0.150	Rank biserial correlation	0.27

**Table 3:** Spearman correlation for sexual dysfunction domains and trunk stability.

Question	Test	Trunk Stability
Desire	Spearman’s rho	0.61
	*p* value	<0.001
	*n*	38
Arousal	Spearman’s rho	0.74
	*p* value	<0.001
	*n*	38
Lubrication	Spearman’s rho	0.72
	*p* value	<0.001
	*n*	38
Orgasm	Spearman’s rho	0.76
	*p* value	<0.001
	*n*	38
Satisfaction	Spearman’s rho	0.72
	*p* value	<0.001
	*n*	38
Pain	Spearman’s rho	0.58
	*p* value	<0.001
	*n*	38

**Table 4:** Intersection point, sexual dysfunction estimators, and *p* values.

	Estimator	SE	95% CI		*p*
			High	Low	
**Interception ^a^**	−2.77	2.08	−7.00	1.46	0.192
**Trunk stability**	40.25	3.77	32.61	47.90	<0.001
**Group: CG** − **StrG**	−5.73	1.98	−9.75	−1.71	0.007
**R^2^**	0.82				
**Durbin–Watson**	1.70				0.298
**Collinearity**	2				
**Shapiro–Wilk**	0.98				0.659

^a^ Reference level. CI = confidence interval; SE = standard error; StrG = stroke group; CG = control group.

## Data Availability

The data presented in this study are available on request from the corresponding authors.
